# A Case of Malignant Melanoma of the Uterine Cervix with Disseminated Metastases throughout the Vaginal Wall

**DOI:** 10.1155/2017/5656340

**Published:** 2017-01-18

**Authors:** Tomoko Noguchi, Nami Ota, Yasushi Mabuchi, Shigetaka Yagi, Sawako Minami, Hisako Okuhira, Yuki Yamamoto, Yasushi Nakamura, Kazuhiko Ino

**Affiliations:** ^1^Department of Obstetrics and Gynecology, Wakayama Medical University, Wakayama, Japan; ^2^Department of Dermatology, Wakayama Medical University, Wakayama, Japan; ^3^Division of Pathology, Department of Clinical Laboratory Medicine, Wakayama Medical University, Wakayama, Japan

## Abstract

Malignant melanoma (MM) in the female genital tract accounts for less than 2% of all melanomas, and the vast majority associated occur in the vulva and vagina. Primary MM of the uterine cervix is extremely rare and its prognosis is very poor. We report a case of primary MM of the cervix with dissemination throughout the vaginal wall. A 66-year-old woman presented with postmenopausal bleeding. Gynecologic examination demonstrated a 2 cm polypoid blackish-pigmented tumor on the cervix with multiple small blackish-pigmented lesions throughout the vaginal wall. Cervical Pap smear cytology showed malignant melanoma. MRI and PET/CT did not detect any distant or lymph node metastases. She underwent radical hysterectomy, pelvic lymphadenectomy, and total vaginectomy. The pathological diagnosis was FIGO stage IIIA primary cervical MM. She received adjuvant chemotherapy with 6 courses of dacarbazine, but 6 months later, multiple lung metastases were detected. Despite 4 courses of anti-PD-1 antibody (nivolumab) treatment, she died of the disease 13 months after surgery.

## 1. Introduction

Malignant melanoma (MM) is a common neoplasm of the skin and mucous membranes. Less than 2% of all MMs occur in the female genital tract [1], and the majority of cases of MM in the female genital tract have been reported in the vulva and vagina [2]. Primary MM of the uterine cervix is extremely rare and its associated prognosis is very poor [3]. Radical hysterectomy with lymphadenectomy is selected in operable cases [4], although there is no consensus regarding standard treatment for this disease. Here, we report a case of primary MM of the cervix with multiple disseminated metastases throughout the vaginal wall, treated with radical hysterectomy and total vaginectomy.

## 2. Case Presentation

**Figure 1:**
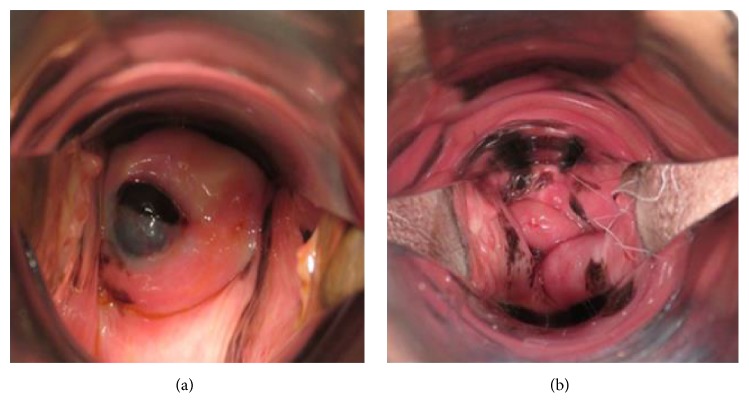
Macroscopic findings demonstrated a 2 cm polypoid blackish-pigmented tumor in the cervix (a). Multiple small blackish-pigmented tumors throughout the vaginal wall spreading to the lower third of the vagina (b).

**Figure 2:**
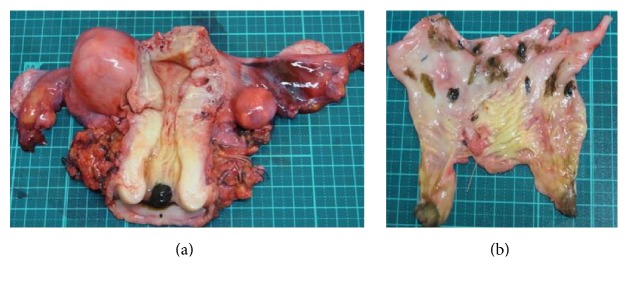
Surgical specimens showed a 2 cm polypoid blackish-pigmented tumor in the uterine cervix (a), and multiple small blackish-pigmented tumors throughout the vaginal wall (b).

**Figure 3:**
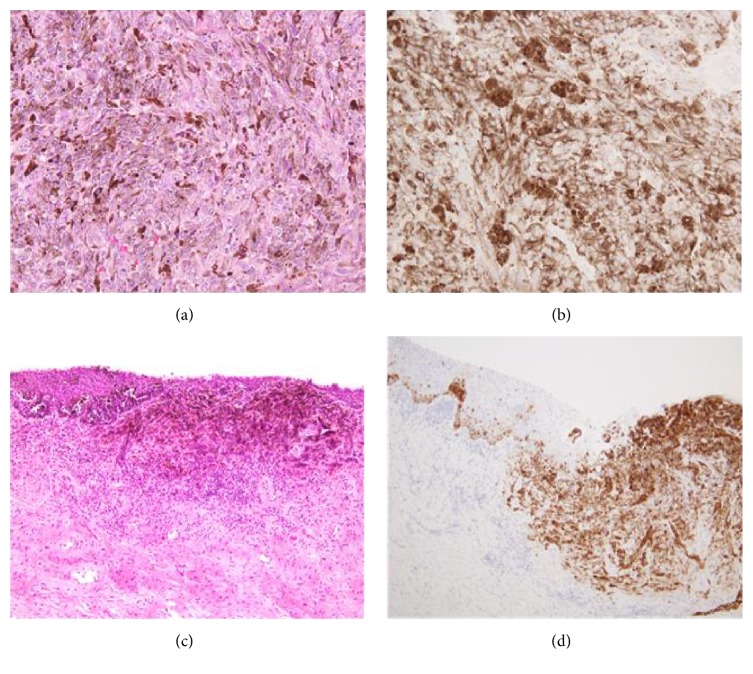
Pathological findings of the cervix revealed spindle-shaped tumor cells showing intracytoplasmic melanin (a) and strong reactivity for melan-A (b). There were similar findings in the vagina (c, d). The invasion depth of the cervical tumor was deeper than that of the vaginal tumors. Magnification: (a) ×400, (b) ×400, (c) ×40, (d) ×100.

A 66-year-old woman presented with postmenopausal bleeding. Gynecologic examination and colposcopic findings revealed a 2 cm polypoid blackish-pigmented tumor in the cervix, and multiple small blackish-pigmented lesions were found throughout the vaginal wall, spreading to the lower third of the vagina (Figure 1). Cervical Pap smear showed MM. MRI and PET/CT did not detect any distant or lymph node metastases. The serum level of 5-SCD, a tumor marker for melanoma, was 5.1 nmol/L (normal level: 1.5*～*8.0). She underwent radical hysterectomy, bilateral salpingooophorectomy, pelvic lymphadenectomy, and total vaginectomy (Figure 2) without any major complications, and optimal surgery was achieved with no residual tumors. The operative time was 333 min, and blood loss was 1335 mL. Pathological examination of the cervix and vaginal wall demonstrated spindle-shaped tumor cells showing intracytoplasmic melanin and strong reactivity for melan-A (Figure 3). The tumor of the cervix was larger than that of the vagina, and the depth of tumor invasion in the cervix was 7 mm, while the depth of invasion of the vaginal lesions was very shallow. The endometrium, bilateral adnexa, lymph nodes, and vaginal stump were free of tumors. Therefore, the final diagnosis was stage IIIA primary cervical melanoma with dissemination to the vaginal wall according to the International Federation of Gynecology and Obstetrics staging system. She received 6 courses of adjuvant chemotherapy with dacarbazine after surgery, but CT six months later showed multiple lung metastases, and she received 4 courses of anti-PD-1 antibody (nivolumab). However, the level of 5-SCD was elevated to 187.9 nmol/L, and CT showed increased lung and bone metastases. She and her family chose palliative care, and she died 13 months after surgery.

## 3. Discussion

Primary MM of the cervix is a rare entity. The incidence of genital tract melanomas has been reported to be 1.6 cases per 1 million females [5]. Most cases of genital tract melanoma occur in the vulva (76.7%) and vagina (19.8%) and more rarely (3–9%) in the cervix [5, 6]. The peak incidence of patients with primary MM of the cervix occurs between 60 and 70 years, and it is likely to present with vaginal discharge, bleeding, or dyspareunia [3, 7, 8]. The diagnosis is usually based on gynecologic examination, colposcopy, and cervical pathology. Cervical Pap smears usually show round or spindle atypical cells containing melanin pigments [9]. Cervical melanoma originates from the melanocytic cells of the cervix [10]. About half of the melanomas are amelanotic [9], and due to the absence of pigmentation, the diagnosis of amelanotic melanoma may be difficult to distinguish from rhabdomyosarcoma, leiomyosarcoma, mixed Müllerian tumor, adenocarcinoma, and poorly differentiated squamous cell carcinoma. Immunostaining is useful for the diagnosis of MM. Protein S100 is considered sensitive and protein HMB 45 is specific to confirm MM, and it is more useful when the two markers are combined [9].

Norris and Taylor [11] proposed four criteria for the diagnosis of primary cervical MM: (1) presence of melanin in the normal cervical epithelium, (2) absence of melanoma elsewhere in the body, (3) demonstration of junctional change in the cervix, and (4) metastases according to the pattern of cervical carcinoma. In this case, the tumor size of the cervix was larger, and the invasion depth of the cervical tumor was deeper than that of the vaginal tumors. Therefore, we conclude that the cervix was the primary site and it disseminated to the vaginal wall.

There is no consensus regarding standard management for primary cervical MM due to its rarity. The most common treatment based on the literature is surgery, including radical hysterectomy coupled with pelvic lymphadenectomy for stage I-II disease or pelvic exenteration for advanced cases [4]. In our case, we selected radical hysterectomy with total vaginectomy. In general, MM in female genital tract easily spreads into the rectum, bladder, and urethral tube. Because there was no evidence of extending to these tracts beyond cervical disease on MRI and PET/CT preoperatively, we avoided pelvic exenteration, considering the age of the patient and her quality of life after surgery. According to Pucceddu's review, of 76 primary cervical MM cases, vaginectomy was performed in only two cases [3]. MM is considered a radio-resistant tumor, and radiotherapy has been used as adjuvant or palliative treatment. Chemotherapy was performed using the same protocol for skin melanoma [12]. Dacarbazine as a single agent is the most commonly used drug, with response rates (RR) of about 15–20%. Combination chemotherapy with cisplatin, vinblastine, and dacarbazine led to RR of 20–35%, but this was not more effective than dacarbazine alone for prolonging survival [13]. There is a lack of evidence on the efficacy of postoperative radiation or chemotherapy. Nivolumab (anti-PD-1) was made available in Japan from July 2014, and it is expected to be effective for MM in the female genital tract. The prognosis associated with primary cervical MM is generally poor because its diagnosis is usually made at an advanced stage. According to a recent review, the 5-year survival is 18.8% for stage I, 11.1% for stage II, and 0% for stages III and IV [3]. In our case, we achieved optimal surgery for primary cervical MM without pelvic exenteration. However, the tumor recurred in the lung and rapidly progressed despite treatment with dacarbazine or nivolumab. Further studies are needed in order to propose standard treatment for primary cervical MM.
